# Guanosine Attenuates Astrocyte-Associated Glutamatergic Dysregulation and Improves Survival in Acute Liver Failure-Induced Hepatic Encephalopathy

**DOI:** 10.3390/metabo16080587

**Published:** 2026-08-18

**Authors:** Pedro Arend Guazzelli, Felipe dos Santos Fachim, Anderson Santos Travassos, Yasmine Nonose, Andréia Silva da Rocha, Francieli Rohden, Fernanda Urruth Fontella, Adriano Martimbianco de Assis, Diogo Onofre Souza

**Affiliations:** 1 Graduate Program in Biological Sciences: Biochemistry, Instituto de Ciências Básicas da Saúde (ICBS), Universidade Federal do Rio Grande do Sul—UFRGS, Porto Alegre 90035-003, RS, Brazilanderson.travassos@gmail.com (A.S.T.);; 2 Graduate Program in Health and Behaviour, Center of Health Science, Universidade Católica de Pelotas—UCPel, Pelotas 96015-560, RS, Brazil; 3 Department of Biochemistry, Universidade Federal do Rio Grande do Sul—UFRGS, Porto Alegre 90035-003, RS, Brazil

**Keywords:** acute liver failure, hepatic encephalopathy, guanosine, neuroprotection, astrocytes, glutamate uptake, cerebrospinal fluid biomarkers

## Abstract

**Background/Objectives:** Acute liver failure (ALF) rapidly induces hepatic encephalopathy (HE), a severe neurological syndrome associated with astrocytic dysfunction and glutamatergic dysregulation. Guanosine (GUO), an endogenous guanine-based nucleoside, has neuroprotective properties, but its effects on astrocyte-associated glutamate regulation in ALF-induced HE remain incompletely understood. This study tested whether GUO attenuates neurological deterioration and glutamatergic dysfunction in an experimental model of ALF-induced HE. **Methods:** Male Wistar rats underwent 92% subtotal hepatectomy and received intraperitoneal GUO (7.5 mg/kg) or saline at prespecified time points after surgery. Neurological severity and survival were monitored for 72 h. Astrocytic morphology was assessed by GFAP immunofluorescence. Cerebrospinal fluid (CSF) albumin, glutamate, and glutamine levels, cortical Na^+^-dependent glutamate uptake, GLAST immunocontent, and the 67 kDa GLT-1 monomer immunocontent were evaluated. **Results:** Subtotal hepatectomy induced progressive neurological impairment, high mortality, GFAP-associated astrocytic remodeling, increased CSF albumin, glutamate, and glutamine levels, and reduced cortical glutamate uptake. GUO attenuated neurological deterioration and increased 72 h survival from 10.5% to 39.0% (log-rank *p* = 0.03). GUO also reduced CSF albumin, glutamate, and glutamine concentrations and improved cortical Na^+^-dependent glutamate uptake without altering GLAST or GLT-1 monomer immunocontent. **Conclusions:** GUO attenuated astrocyte-associated glutamatergic dysregulation and improved survival in ALF-induced HE. These findings support further mechanistic and translational investigation of GUO as an experimental modulator of astrocyte-associated glutamate handling in ALF-induced HE.

## 1. Introduction

Acute liver failure (ALF) is the rapid loss of hepatic function in a person without pre-existing liver disease and is characterized by coagulopathy and hepatic encephalopathy (HE), with jaundice and multiorgan dysfunction frequently present [1,2]. Major etiologies include drug-induced liver injury, viral hepatitis, ischemic injury, and autoimmune liver disease [3,4]. Acetaminophen toxicity predominates in several Western cohorts, whereas viral etiologies remain important in other regions [5,6]. Despite advances in critical care and liver transplantation, ALF remains associated with substantial mortality because of complications including cerebral edema, intracranial hypertension, infection, circulatory failure, renal dysfunction, and bleeding [7,8,9].

HE is the principal neurological complication of ALF and encompasses a continuum of neuropsychiatric impairment [10,11,12]. Mild or early HE (grades I–II) may present with altered attention, sleep–wake disturbance, mood or behavioral changes, disorientation, tremor, and reduced psychomotor activity. Severe HE (grades III–IV) is characterized by marked somnolence or stupor, loss of protective reflexes, coma, cerebral edema, intracranial hypertension, brain herniation, and death and often requires intensive neurocritical care and urgent evaluation for liver transplantation [1,12,13,14,15,16]. This clinical progression highlights the need for interventions that act before irreversible neurological deterioration occurs.

The pathophysiology of HE is multifactorial, with hyperammonemia playing a central role [17,18]. Elevated ammonia is associated with intracranial hypertension [19,20], and its neurotoxicity is amplified by systemic inflammation and other circulating toxins [21,22]. Within the brain, ammonia-related injury promotes astrocytic glutamine accumulation and swelling, oxidative and nitrosative stress, neuroinflammation, energetic failure, altered blood–brain barrier and neurovascular function, and dysregulation of excitatory and inhibitory neurotransmission [18,23]. Astrocytes are particularly vulnerable; accordingly, HE has been described as a gliopathy [17,18,24,25,26]. These cells regulate extracellular glutamate through the transporters GLAST and GLT-1 and subsequently metabolize glutamate and ammonia to glutamine through glutamine synthetase (GS) [27,28,29,30,31,32]. Hyperammonemia can impair glutamate uptake and disrupt GS-dependent metabolism [33,34,35]. Experimental studies targeting NMDA receptor signaling or GS activity have also demonstrated effects on survival in acute liver failure models [33,34,35].

Experimental HE produces profound alterations in the glutamatergic system. Reduced astrocytic glutamate uptake increases extracellular glutamate and may contribute to neuronal hyperexcitability and excitotoxicity [28,31,36]. However, glutamate uptake, transporter abundance, intracellular glutamate metabolism, and GS activity are distinct biological processes and should not be considered interchangeable. Assessing these components separately is important for defining which aspects of astrocyte-associated glutamatergic regulation are modified during ALF and by candidate treatments.

GUO is an endogenous guanine-based nucleoside with neuroprotective and neuromodulatory effects in several experimental models of brain injury [37,38]. Previous studies from our group have demonstrated that GUO improves neurological, behavioral, neurochemical, and redox outcomes in experimental hyperammonemia and chronic HE [31,39]. Nevertheless, whether GUO improves survival and preserves astrocyte-associated glutamate handling during rapidly progressive ALF-induced HE has not been fully established.

Accordingly, this study investigated the effects of GUO in a rat model of ALF-induced HE produced by 92% subtotal hepatectomy. We hypothesized that GUO would attenuate neurological deterioration and improve survival in association with reduced astrocytic reactivity and improved cortical glutamate uptake. Neurological outcomes, survival, GFAP-associated astrocytic morphology, CSF albumin, glutamate and glutamine concentrations, cortical Na^+^-dependent glutamate uptake, GLAST immunocontent, and GLT-1 monomer immunocontent were evaluated. Because GS expression/activity and glutamate-glutamine flux were not directly measured, the study was designed to assess selected components of astrocyte-associated glutamatergic regulation rather than the entire glutamate-glutamine cycle.

## 2. Methods

### 2.1. Reagents

Unless otherwise specified, reagents were purchased from Sigma-Aldrich (St. Louis, MO, USA). The radiolabeled tracer used for the glutamate-uptake assay was L-[3,4-^3^H]glutamate (PerkinElmer, Boston, MA, USA). Optiphase “Hisafe” 3 scintillation liquid was obtained from PerkinElmer (Boston, MA, USA). Protein concentrations were measured with the BCA Protein Assay Kit (catalog no. 23227; Thermo Fisher Scientific, Rockford, IL, USA), using bovine serum albumin as the standard.

### 2.2. Animals, Experimental Cohorts, Allocation, Blinding, and Endpoint-Specific Sample Sizes

Male Wistar rats (90 days old) were obtained from the Animal House of the Department of Biochemistry, ICBS, Federal University of Rio Grande do Sul, Porto Alegre, Brazil. Animals were housed four per cage under a 12 h light/dark cycle (lights on 07:00–19:00) at 22 ± 1 °C, with ad libitum access to water and standard chow (SUPRA, Porto Alegre, Brazil). The experimental protocol (project no. 29468) was approved on 1 March 2019 by the Ethics Committee for Animal Research of the Federal University of Rio Grande do Sul, Porto Alegre, Brazil, and was conducted in accordance with the NIH Guide for the Care and Use of Laboratory Animals (NIH Publication No. 85-23, revised 1996). Humane endpoints included intolerable pain, uncontrolled hemorrhage, or seizures during surgery; affected animals would have been euthanized by exsanguination under anesthesia. Three animals died during surgery in the overall experimental series (sham, *n* = 1; hepatectomy, *n* = 2). Only male rats were used to maintain comparability with previous studies using this model; the absence of female animals is acknowledged as a limitation [40,41].

Animals were assigned to four groups: Sham + Saline, Sham + GUO, Hepatectomy + Saline, and Hepatectomy + GUO. The archived experimental records describe group allocation as randomized, but the method used to generate and implement the allocation sequence was not retained. Neurological scoring was performed by an investigator blinded to treatment allocation. Blinding was not documented for surgery, sample processing, biochemical assays, image acquisition or selection, GFAP morphometry, Western-blot densitometry, or statistical analysis; therefore, no blinding is claimed for those stages. No formal a priori sample-size or power calculation was performed. Sample sizes were determined based on previous studies from our group using the same 92% subtotal-hepatectomy model, the expected mortality of the model, and the tissue or sample requirements of the prespecified assays. The animal was the biological experimental unit and the unit of statistical inference. Measurements from multiple sections, fields, slices, or assay wells from the same animal were averaged to obtain one animal-level value and were not treated as independent biological replicates.

Animals were allocated to separate experimental cohorts according to the planned outcomes. The neurological-severity and survival cohort included 38 animals in the Hepatectomy + Saline group and 41 animals in the Hepatectomy + GUO group. A separate cohort was euthanized 24 h after surgery for CSF, biochemical, histological, and Western-blot analyses. Because of the high mortality inherent in the 92% subtotal-hepatectomy model, endpoint-specific sample and tissue availability, and assay requirements, the final number of animals differed among outcomes. For the Sham + Saline, Sham + GUO, Hepatectomy + Saline, and Hepatectomy + GUO groups, respectively, the final biological sample sizes were 18/18/20/18 for GFAP primary-process analysis; 18/11/18/18 for GFAP secondary-process analysis; 12/8/10/9 for CSF albumin; 5/6/6/6 for CSF glutamate; 6/6/6/5 for CSF glutamine; 10/5/9/8 for Na^+^-dependent glutamate uptake; and 4/4/3/3 for both GLAST and GLT-1 monomer immunocontent. Detailed animal flow, recoverable surgical or precollection losses, postcollection exclusions or unavailable observations, nested measurements and experimental units are presented in Supplementary Table S1.

### 2.3. Guanosine Preparation and Administration

Guanosine was prepared at 7.5 mg/mL in sterile 0.9% NaCl. Animals assigned to GUO received intraperitoneal injections of 1 mL/kg (7.5 mg/kg per dose), whereas control animals received an equal volume of saline. In the 24 h terminal cohort, GUO or saline was administered immediately after surgery (0 h) and at 3, 6, and 12 h. In the neurological-severity and survival cohort, additional doses were administered at 24, 36, 48, and 60 h. No injection was administered at 72 h.

The selected GUO dose and administration schedule were based on previous work from our group and preliminary dose-ranging experiments [39].

### 2.4. Subtotal Hepatectomy and Sham Surgery

The 92% subtotal-hepatectomy model was performed as previously described [28,36,42,43,44,45]. Anesthesia was induced and maintained with 3% isoflurane in oxygen at 1 L/min. After antisepsis with povidone-iodine, a midline laparotomy was performed. Hepatic ligaments and adhesions were divided, the pedicles of the anterior and right lobes were ligated with 4-0 silk, and the corresponding lobes were resected, leaving only the omental lobes (approximately 8% of liver mass) functional [46]. Sham-operated animals underwent the same anesthesia, laparotomy, and manipulation without pedicle ligation or liver resection.

After closure of the abdominal wall, 0.8 mL lidocaine was administered at the wound margins for local analgesia. Animals recovered for 30 min in a heated chamber (25 °C) before returning to their home cages. To reduce postoperative hypoglycemia, animals received 20% glucose in drinking water for 24 h and intraperitoneal injections of the same glucose solution (2 mL/kg) at 0, 6, and 12 h [47,48]. Neurological assessments were initiated only after recovery from anesthesia, and sham animals underwent the same anesthetic exposure and postoperative procedures.

This ALF model has previously been used by our group to characterize alterations in brain energy metabolism, glial reactivity, neurochemistry, behavior, and electroencephalographic activity [28,36].

Endpoint-specific biological sample sizes and animal-flow information are summarized in Supplementary Table S1.

### 2.5. Neurological Severity and Survival

The neurological grading scale was applied as previously described [45] at 0, 3, 6, 12, 24, 36, 48, 60, and 72 h after surgery. Grade 0 indicated spontaneous locomotion and exploratory activity; grade I, immobility without abnormal reflexes; grade II, impaired locomotion with preserved rapid righting; grade III, inability to right with preserved corneal reflexes; grade IV, abnormal corneal or pain responses; and grade V, death. Neurological scoring was blinded to treatment. Because the archived dataset retained group-level grade counts at each time point but not animal-level identifiers linking repeated observations, longitudinal within-animal modeling was not possible. Exploratory Mann–Whitney comparisons were therefore performed between the two hepatectomized groups at 6, 12, 24, 36, 48, 60, and 72 h, with death coded as grade V and Holm adjustment across time points. Kaplan–Meier analysis was performed separately using the complete animal-level time-to-event data. At 24 and 48 h, the analyses were based on the available categorized animals, as detailed in Supplementary Table S1.

### 2.6. Biochemical and Histological Analyses

Unless otherwise stated, biochemical and histological analyses were performed 24 h after surgery. The final biological n and technical-replicate structure for each assay are reported in Supplementary Table S1.

#### 2.6.1. Cerebrospinal Fluid Collection

CSF was collected 24 h after surgery as previously described [28,39,49]. Animals were briefly anesthetized with 3% isoflurane and positioned in a stereotaxic frame. Approximately 150 µL of CSF was obtained by direct cisterna magna puncture with a 29-gauge insulin syringe, centrifuged at 10,000× *g* for 10 min, and the supernatant was stored at −80 °C. Only samples with sufficient volume for the specified assay were included; endpoint-specific availability is reported in Supplementary Table S1.

#### 2.6.2. CSF Albumin Quantification by HPLC-FLD

CSF albumin was measured by high-performance liquid chromatography with fluorescence detection (HPLC-FLD) using a Shimadzu LC system (Shimadzu Corporation, Kyoto, Japan) equipped with an LC-20AT pump, DGU-14A degasser, CTO-10A column oven, and RF-20A fluorescence detector. LCsolution software version 1.25 (Shimadzu Corporation, Kyoto, Japan) was used for acquisition and processing. Excitation and emission wavelengths were 278 and 335 nm, respectively. Separation was performed on a ZORBAX SB-C18 reversed-phase column (5 µm, 250 × 4.6 mm; Agilent Technologies, Santa Clara, CA, USA). Mobile phases were water containing 0.1% formic acid (A) and acetonitrile (B), with 35% B from 0 to 5 min, 30% B from 5 to 10 min, and 35% B from 10 to 17 min, at 0.7 mL/min. Albumin standards (0.1, 0.5, 1, 10, 50, and 100 µg/mL) were prepared daily. A 10 µL aliquot of CSF was mixed with 40 µL acetonitrile, vortexed, transferred to a conical vial, and 10 µL was injected. Albumin was quantified from the calibration curve and reported in µg/mL.

#### 2.6.3. CSF Glutamate and Glutamine Quantification

CSF glutamate and glutamine were quantified by reversed-phase HPLC with precolumn o-phthalaldehyde derivatization, as previously described [45,50]. A Supelcosil LC-18 column (5 µm, 250 × 4.6 mm; Supelco, Bellefonte, PA, USA) was used in a Shimadzu liquid chromatograph with a 50 µL loop and 40 µL injection volume. For derivatization, 20 µL of sample was mixed with 100.5 µL OPA solution (5.4 mg OPA in 1 mL 0.2 M sodium borate, pH 9.5) and 25.5 µL 4% mercaptoethanol for 3 min. The flow rate was 1.4 mL/min and the column temperature was 24 °C. Buffer A was 0.04 mol/L sodium dihydrogen phosphate monohydrate, pH 5.5, containing 80% methanol; buffer B was 0.01 mol/L sodium dihydrogen phosphate monohydrate, pH 5.5, containing 20% methanol. The proportion of buffer B was 100% at 0.1 min, 90% at 10 min, 48% at 15 min, and 100% at 60 min. Fluorescence was monitored at 360 nm excitation and 455 nm emission. Glutamate and glutamine were identified by retention time and quantified by peak area using an amino-acid standard mixture for calibration and quality control. Concentrations are reported in µM.

#### 2.6.4. Na^+^-Dependent Glutamate Uptake in Cerebral-Cortex Slices

Na^+^-dependent glutamate uptake was measured in frontoparietal cortical slices as previously described [31,51]. Immediately after decapitation, the cortex was dissected on ice and 0.2 mm slices were prepared with a McIlwain Tissue Chopper (Mickle Laboratory Engineering Co., Ltd., Guildford, UK). Slices were immersed in ice-cold Hank’s balanced salt solution (HBSS; in mM: 137 NaCl, 0.63 Na_2_HPO_4_, 4.17 NaHCO_3_, 5.36 KCl, 0.44 KH_2_PO_4_, 1.26 CaCl_2_, 0.41 MgSO_4_, 0.49 MgCl_2_, and 1.11 glucose; pH 7.2) and preincubated at 37 °C for 15 min. Uptake was initiated by adding L-[3,4-^3^H]glutamate (PerkinElmer, Boston, MA, USA) at 0.33 µCi/mL to a final total glutamate concentration of 100 µM and was stopped after 7 min by two washes with 1 mL ice-cold HBSS. Slices were solubilized overnight in 0.5 N NaOH. Na^+^-independent uptake was measured at 4 °C in Na^+^-free medium in which N-methyl-D-glucamine replaced NaCl; Na^+^-dependent uptake was calculated as total minus Na^+^-independent uptake. Radioactivity was measured with a Hidex 300 SL liquid scintillation counter (Hidex Oy, Turku, Finland). Protein content was determined by BCA assay, and uptake was expressed as nmol glutamate/mg protein/min.

#### 2.6.5. GFAP Immunofluorescence and Astrocyte Morphometry

GFAP immunofluorescence was performed to assess astrocytic morphology in the frontoparietal cortex 24 h after surgery. Animals were anesthetized with 3% isoflurane and euthanized by decapitation. Brains were immersion-fixed for 24 h in 4% paraformaldehyde in phosphate-buffered saline (PBS; pH 7.4), cryoprotected sequentially in 15% and 30% sucrose until the tissue sank, frozen at −20 °C, and sectioned coronally at 30 µm with a cryostat (SLEE Medical GmbH, Mainz, Germany), beginning approximately +2.20 mm from bregma. Free-floating sections were washed in PBS, permeabilized in PBS containing Triton X-100, and incubated for 24 h at 4 °C with rabbit polyclonal anti-GFAP antibody (Z0334, 1:500; Dako, Glostrup, Denmark). After PBS washes, sections were incubated for 2 h with Alexa Fluor 555 goat anti-rabbit secondary antibody (1:1000; Invitrogen, Carlsbad, CA, USA), washed, mounted, and imaged using an Olympus FV1000 confocal microscope (Olympus Corporation, Tokyo, Japan). Images were acquired as 8-bit grayscale files using identical acquisition settings across groups. For each animal, two to three coronal sections were analyzed, with three non-overlapping fields acquired per section from standardized regions of the frontoparietal cortex using a 40× objective. Between 15 and 30 GFAP-positive astrocytes were evaluated per animal. Images were analyzed using ImageJ version 1.54f (National Institutes of Health, Bethesda, MD, USA). A primary process was defined as a GFAP-positive process emerging directly from the astrocyte soma, and a secondary process as a branch arising from a primary process. Cell-level measurements from the same animal were averaged to generate a single animal-level value for each outcome.

#### 2.6.6. Western Blotting

Cortical samples collected 24 h after surgery were homogenized in ice-cold lysis buffer containing 4% SDS, 2 mM EDTA, and 50 mM Tris-HCl (pH 6.8). Protein concentrations were measured using the BCA assay, and 10 µg of protein per lane was separated by 10% SDS-PAGE with a 4% stacking gel and transferred to nitrocellulose membranes. Membranes were blocked with 5% skim milk and incubated overnight at 4 °C with rabbit polyclonal anti-GLAST (GLAST11-A, 1:5000; Alpha Diagnostic International, San Antonio, TX, USA) or rabbit polyclonal anti-GLT-1 (GLT11-A, 1:5000; Alpha Diagnostic International). An anti-β-tubulin antibody (1:1000; Cell Signaling Technology, Danvers, MA, USA) was used as the loading control. After washing, membranes were incubated for 1 h with the appropriate horseradish peroxidase-conjugated anti-rabbit or anti-mouse IgG secondary antibody (1:7000). Chemiluminescent signals were developed using the Amersham ECL Prime Western blotting Detection Reagent (RPN2236; Cytiva, Marlborough, MA, USA) and acquired using an ImageQuant LAS 4010 system (GE Healthcare, Chicago, IL, USA). Densitometric analysis was performed using ImageQuant TL 8.1. GLAST was quantified at approximately 55 kDa. For GLT-1, only the monomeric band at approximately 67 kDa was quantified; the higher-molecular-weight immunoreactive bands at approximately 134 and 201 kDa were not included in the analysis. Target-protein signals were normalized to β-tubulin. The biological sample sizes were Sham + Saline (*n* = 4), Sham + GUO (*n* = 4), Hepatectomy + Saline (*n* = 3), and Hepatectomy + GUO (*n* = 3). Each lane corresponded to one animal, and all available lanes were included in the densitometric analyses. No lane rearrangement or splicing was performed.

### 2.7. Statistical Analysis

The animal was the experimental unit. Technical or nested measurements from the same animal were averaged before inferential analysis. For continuous outcomes, residual normality was evaluated using Q–Q plots and the Shapiro–Wilk test, whereas homogeneity of variance was assessed using the Brown–Forsythe test. Outcomes measured across the four independent groups were analyzed by two-way ANOVA with surgical status (sham versus hepatectomy) and treatment (saline versus GUO) as fixed factors. Type III sums of squares were used because group sizes were unequal. CSF glutamate, CSF glutamine, and cortical Na^+^-dependent glutamate uptake were log10-transformed before analysis; descriptive values are presented in the original scale. Prespecified simple-effects comparisons were adjusted using the Šidák procedure. Partial eta squared (ηp^2^) was used as the effect-size measure, and 95% confidence intervals are reported for principal planned contrasts. Primary and secondary GFAP-process counts were analyzed using a two-factor aligned rank transform, followed by Holm-adjusted planned pairwise comparisons. Neurological grades were treated as ordinal data; the two hepatectomized groups were compared at 6, 12, 24, 36, 48, 60, and 72 h using Mann–Whitney tests with Holm adjustment across the seven time points, with death coded as grade V. These time-point analyses are exploratory because individual longitudinal identifiers were not retained in the archived grade-count table. Survival was estimated by Kaplan–Meier analysis and compared with the log-rank (Mantel–Cox) test. Continuous outcomes and GFAP-process counts are presented as mean ± SEM. Neurological-grade distributions are displayed as stacked animal counts by ordinal category. Analyses were performed in GraphPad Prism 10.0, SPSS 27.0, and Python 3.13 using statsmodels 0.14.6 and SciPy 1.17.0. All tests were two-sided, and *p* < 0.05 was considered statistically significant.

## 3. Results

**Figure 1:**
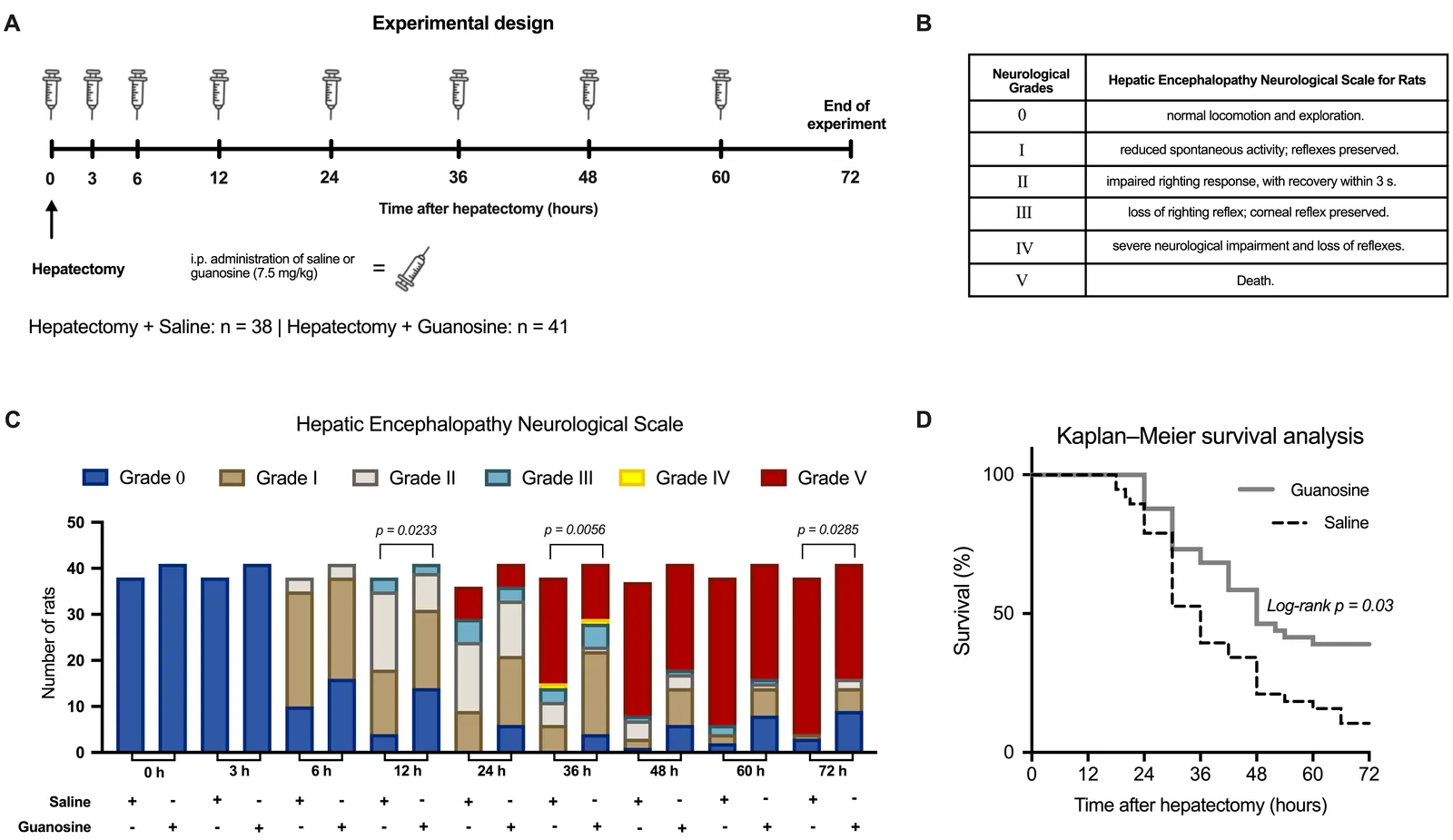
Effects of subtotal hepatectomy and guanosine treatment on neurological progression and survival. (**A**) Experimental timeline showing intraperitoneal (i.p.) administration of saline or guanosine (GUO; 7.5 mg/kg) immediately after surgery and at 3, 6, 12, 24, 36, 48, and 60 h. Neurological assessments were performed at 0, 3, 6, 12, 24, 36, 48, 60, and 72 h after surgery. (**B**) Hepatic encephalopathy neurological grading scale, ranging from grade 0, indicating normal spontaneous locomotion and exploratory activity, to grade V, indicating death. (**C**) Distribution of hepatectomized animals across neurological grades at each assessment time point. Neurological grades were compared exploratorily between the Hepatectomy + Saline and Hepatectomy + GUO groups at 6, 12, 24, 36, 48, 60, and 72 h using Mann–Whitney tests with Holm adjustment across the seven time points. Adjusted *p*-values for the significant comparisons are shown in the panel. (**D**) Kaplan–Meier survival curves for hepatectomized animals treated with saline (*n* = 38) or GUO (*n* = 41). Survival distributions were compared using the Mantel–Cox log-rank test. At 72 h, survival was 10.5% in the Hepatectomy + Saline group and 39.0% in the Hepatectomy + GUO group (log-rank *p* = 0.03).

The 92% subtotal hepatectomy produced progressive neurological deterioration and high mortality (Figure 1). The neurological and survival cohort included 38 Hepatectomy + Saline and 41 Hepatectomy + GUO animals. Exploratory Mann–Whitney comparisons of the ordinal grades, with Holm adjustment across the seven analyzed time points, showed lower neurological severity after GUO at 12 h (U = 1058.0, adjusted *p* = 0.0233), 36 h (U = 1100.5, adjusted *p* = 0.0056), and 72 h (U = 994.0, adjusted *p* = 0.0285); adjusted comparisons at 6, 24, 48, and 60 h were not significant. At 24 and 48 h, the exploratory comparisons were based on the available categorized animals, as detailed in Supplementary Table S1. The complete animal-level time-to-event dataset was used for survival analysis. By 72 h, 34 of 38 saline-treated hepatectomized animals had died, corresponding to 10.5% survival, whereas 25 of 41 GUO-treated hepatectomized animals had died, corresponding to 39.0% survival. Kaplan–Meier curves differed significantly (log-rank Mantel–Cox *p* = 0.03).

**Figure 2:**
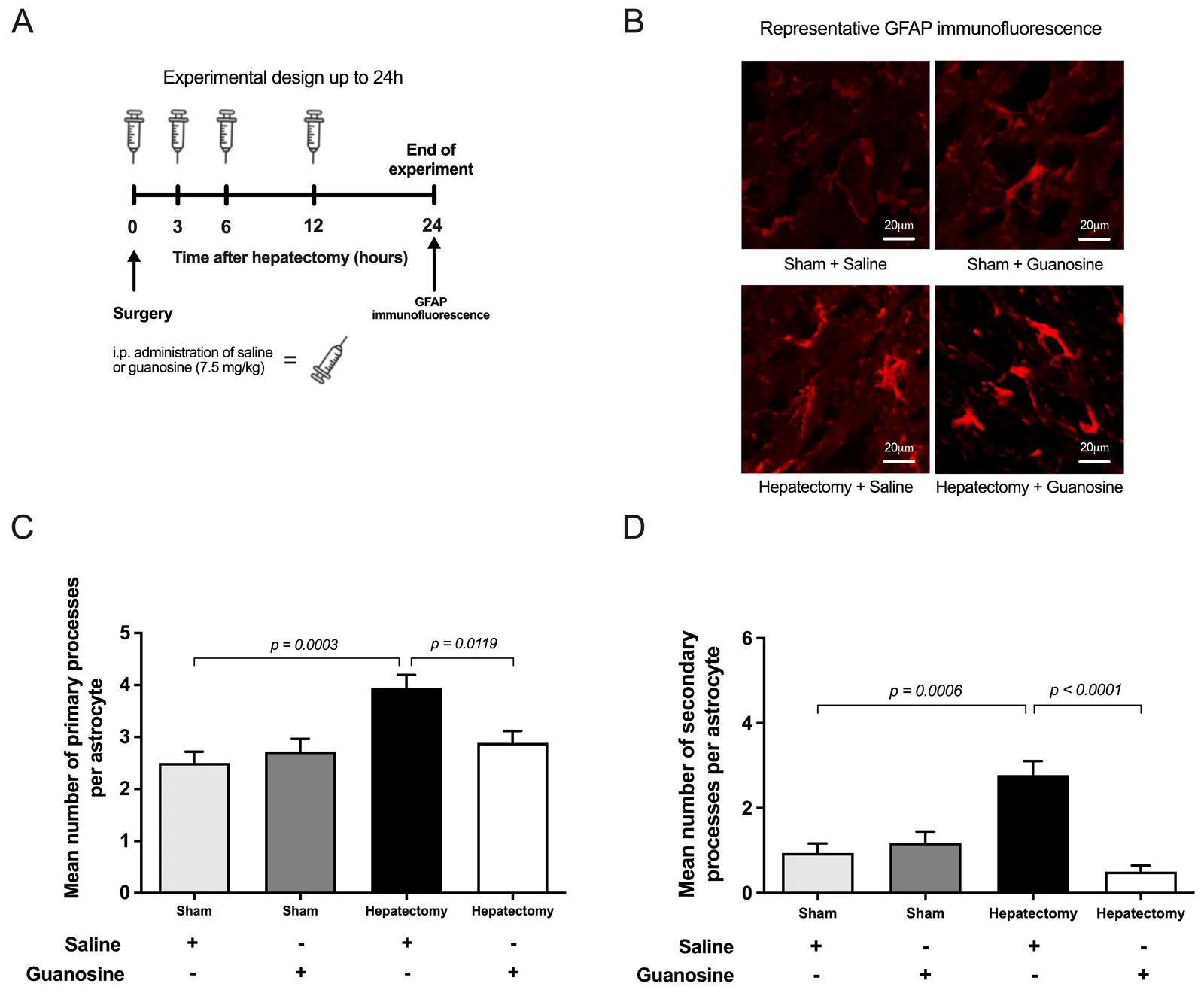
Effects of subtotal hepatectomy and guanosine treatment on GFAP-associated astrocytic morphology in the frontoparietal cortex. (**A**) Experimental timeline showing intraperitoneal (i.p.) administration of saline or guanosine (GUO; 7.5 mg/kg) immediately after surgery and at 3, 6, and 12 h, followed by tissue collection at 24 h. (**B**) Representative confocal immunofluorescence images of GFAP-positive astrocytes in the frontoparietal cortex. Scale bars = 20 µm. (**C**) Mean number of primary processes per astrocyte and (**D**) mean number of secondary processes per astrocyte. Biological sample sizes for the Sham + Saline, Sham + GUO, Hepatectomy + Saline, and Hepatectomy + GUO groups, respectively, were *n* = 18, 18, 20, and 18 for primary-process analysis and *n* = 18, 11, 18, and 18 for secondary-process analysis. Each value represents one animal. Data are presented in the original measurement scale as mean ± SEM. Outcomes were analyzed using a two-factor aligned rank transform, followed by Holm-adjusted planned pairwise comparisons. The adjusted *p*-values are shown in the panels.

At 24 h, GFAP immunofluorescence was used to evaluate astrocytic morphology in the frontoparietal cortex (Figure 2). Each value represented one animal. For primary-process analysis, the biological sample sizes were 18, 18, 20, and 18 in the Sham + Saline, Sham + GUO, Hepatectomy + Saline, and Hepatectomy + GUO groups, respectively. Aligned-rank-transform analysis showed significant effects of surgery (F(1,70) = 21.52, *p* < 0.0001), treatment (F(1,70) = 5.87, *p* = 0.0180), and the surgery × treatment interaction (F(1,70) = 12.81, *p* = 0.0006). Holm-adjusted comparisons showed more primary processes after hepatectomy in saline-treated animals (adjusted *p* = 0.0003) and fewer primary processes after GUO in hepatectomized animals (adjusted *p* = 0.0119); GUO had no significant effect in sham animals. For secondary-process analysis, the biological sample sizes were 18, 11, 18, and 18, respectively. Significant effects of surgery (F(1,61) = 9.95, *p* = 0.0025), treatment (F(1,61) = 13.82, *p* = 0.0004), and surgery × treatment interaction (F(1,61) = 21.27, *p* < 0.0001) were observed. Hepatectomy increased secondary processes in saline-treated animals (adjusted *p* = 0.0006), whereas GUO reduced secondary processes in hepatectomized animals (adjusted *p* < 0.0001); GUO did not significantly alter secondary processes in sham animals.

**Figure 3:**
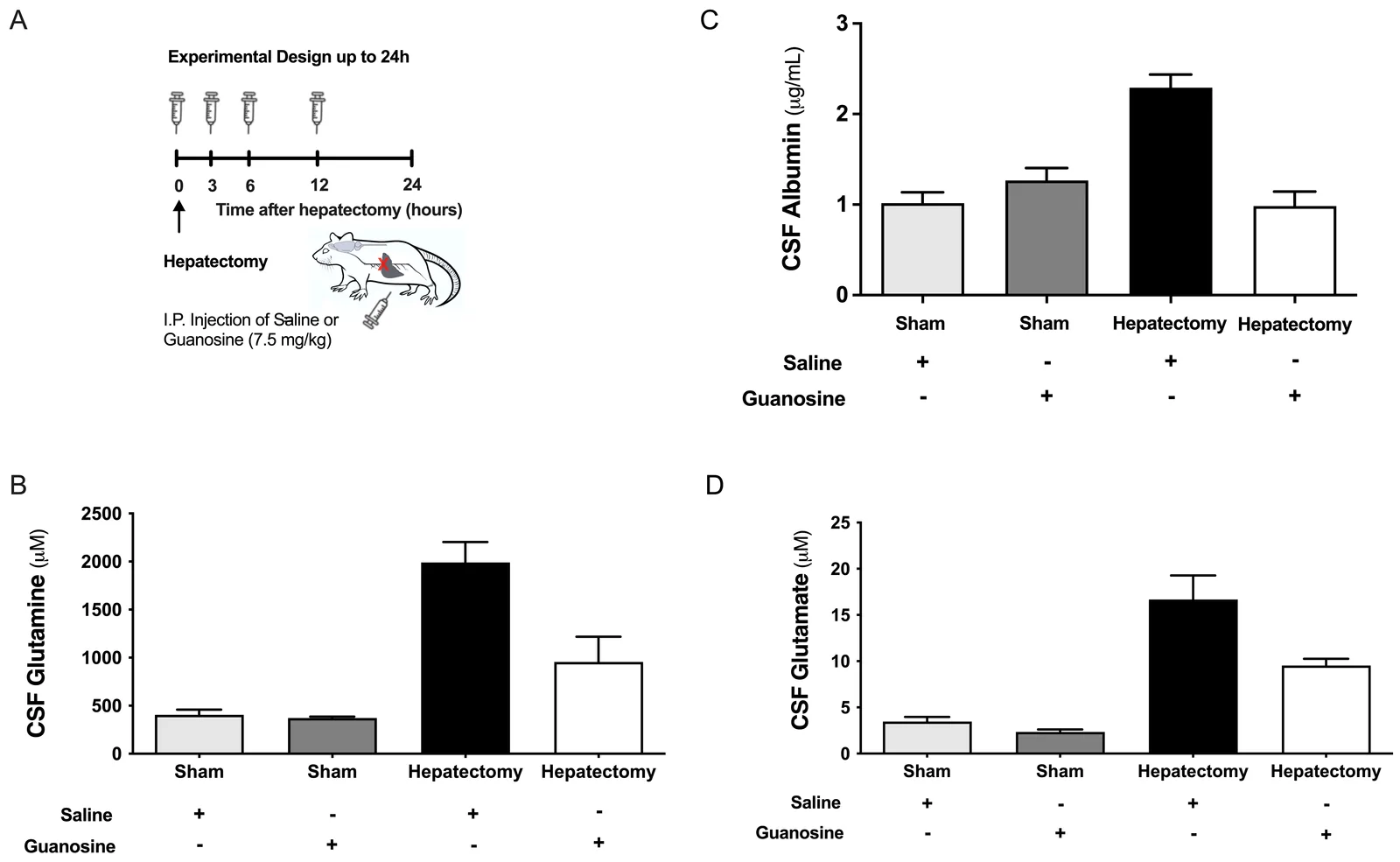
Effects of subtotal hepatectomy and guanosine treatment on cerebrospinal fluid biomarkers 24 h after surgery. (**A**) Experimental timeline showing intraperitoneal (i.p.) administration of saline or guanosine (GUO; 7.5 mg/kg) immediately after surgery and at 3, 6, and 12 h, followed by cerebrospinal fluid collection at 24 h. (**B**) Cerebrospinal fluid albumin concentration, (**C**) glutamate concentration, and (**D**) glutamine concentration. Biological sample sizes for the Sham + Saline, Sham + GUO, Hepatectomy + Saline, and Hepatectomy + GUO groups, respectively, were *n* = 12, 8, 10, and 9 for albumin; *n* = 5, 6, 6, and 6 for glutamate; and *n* = 6, 6, 6, and 5 for glutamine. Each value represents one animal. Data are presented in the original measurement scale as mean ± SEM. Glutamate and glutamine values were log10-transformed before analysis, whereas albumin was analyzed in the original scale. Outcomes were analyzed using Type III two-way ANOVA with surgery and treatment as fixed factors, followed by Šidák-adjusted planned comparisons. The adjusted *p*-values are shown in the panels.

CSF albumin, glutamate, and glutamine concentrations were evaluated 24 h after surgery (Figure 3B–D). Panel B presents albumin (µg/mL), panel C presents glutamate (µM), and panel D presents glutamine (µM). The final biological sample sizes in the Sham + Saline, Sham + GUO, Hepatectomy + Saline, and Hepatectomy + GUO groups, respectively, were 12, 8, 10, and 9 animals for albumin; 5, 6, 6, and 6 for glutamate; and 6, 6, 6, and 5 for glutamine. Glutamate and glutamine values were log10-transformed before factorial analysis, whereas descriptive values are shown in the original units. For CSF albumin, significant effects of surgery (F(1,35) = 12.39, *p* = 0.0012, ηp^2^ = 0.261), treatment (F(1,35) = 14.04, *p* = 0.0006, ηp^2^ = 0.286), and the surgery × treatment interaction (F(1,35) = 30.47, *p* < 0.0001, ηp^2^ = 0.465) were observed. In saline-treated animals, hepatectomy increased CSF albumin (Šidák-adjusted *p* < 0.0001). In hepatectomized animals, GUO reduced albumin by 1.31 µg/mL compared with saline (95% CI −1.71 to −0.90; Šidák-adjusted *p* < 0.0001), whereas GUO did not significantly alter albumin in sham-operated animals (adjusted *p* = 0.387). For CSF glutamate, two-way ANOVA showed significant effects of surgery (F(1,19) = 153.41, *p* < 0.0001, ηp^2^ = 0.890) and treatment (F(1,19) = 13.75, *p* = 0.0015, ηp^2^ = 0.420), but no significant surgery × treatment interaction (F(1,19) = 0.31, *p* = 0.582, ηp^2^ = 0.016). Hepatectomy increased glutamate in saline-treated animals (Šidák-adjusted *p* < 0.0001). In hepatectomized animals, GUO reduced glutamate relative to saline (geometric mean ratio = 0.60, 95% CI 0.42–0.85; Šidák-adjusted *p* = 0.0119); the corresponding comparison in sham animals was not significant after adjustment (*p* = 0.0832). For CSF glutamine, significant effects of surgery (F(1,19) = 42.64, *p* < 0.0001, ηp^2^ = 0.692), treatment (F(1,19) = 7.51, *p* = 0.0130, ηp^2^ = 0.283), and the surgery × treatment interaction (F(1,19) = 6.12, *p* = 0.0230, ηp^2^ = 0.244) were observed. Hepatectomy increased glutamine in saline-treated animals (Šidák-adjusted *p* < 0.0001). In hepatectomized animals, GUO reduced glutamine relative to saline (geometric mean ratio = 0.40, 95% CI 0.23–0.68; Šidák-adjusted *p* = 0.0038), whereas no effect was detected in sham-operated animals (adjusted *p* = 0.977). Because CSF albumin was not accompanied by a direct permeability assay or matched serum albumin measurement, it is interpreted as an indirect marker of barrier-related alterations rather than proof of blood–brain barrier disruption.

**Figure 4:**
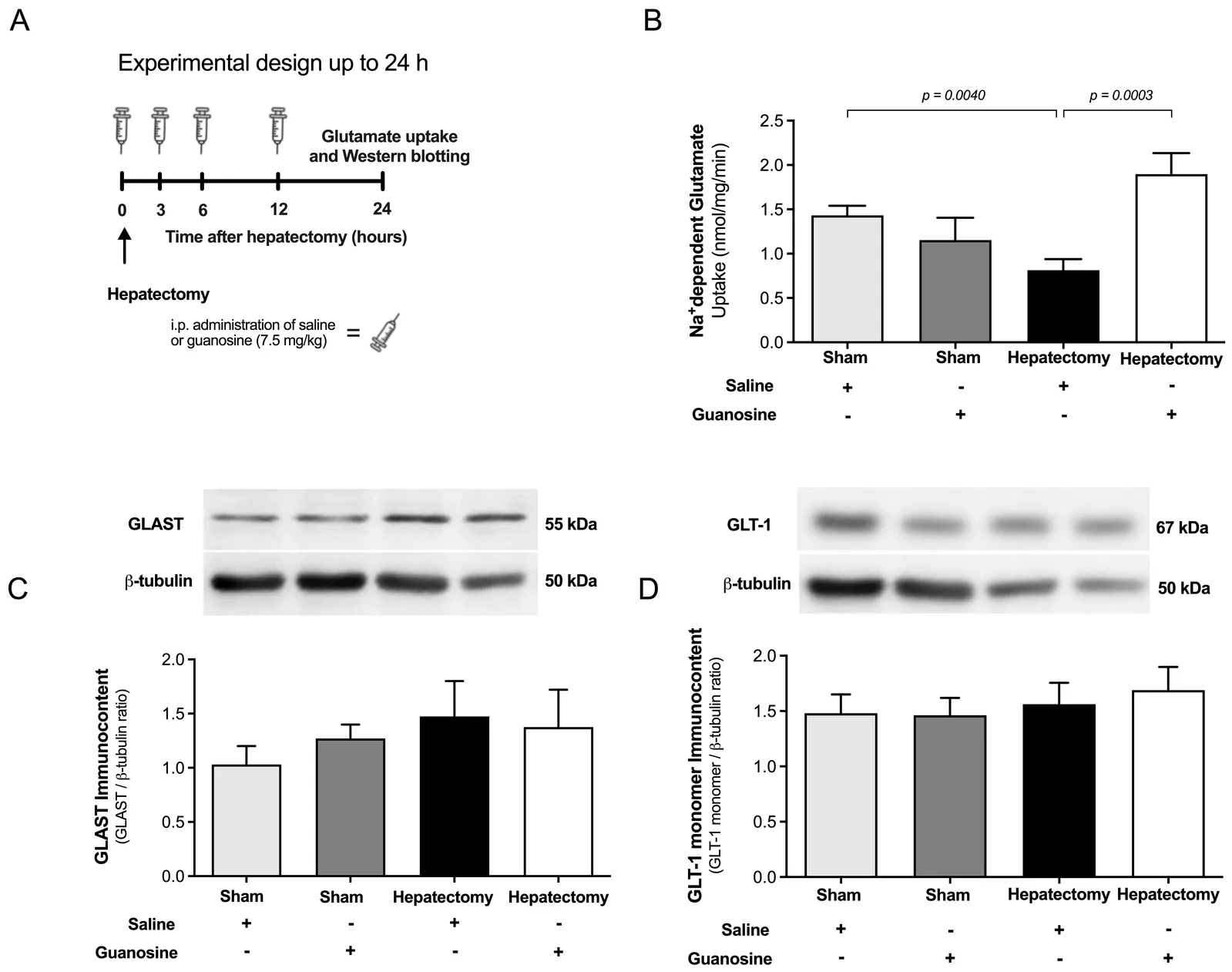
Effects of subtotal hepatectomy and guanosine treatment on cortical Na^+^-dependent glutamate uptake and glutamate-transporter immunocontent. (**A**) Experimental timeline showing intraperitoneal (i.p.) administration of saline or guanosine (GUO; 7.5 mg/kg) immediately after surgery and at 3, 6, and 12 h, followed by tissue collection at 24 h. (**B**) Na^+^-dependent glutamate uptake in frontoparietal cortical slices, expressed as nmol glutamate/mg protein/min. Biological sample sizes for the Sham + Saline, Sham + GUO, Hepatectomy + Saline, and Hepatectomy + GUO groups, respectively, were *n* = 10, 5, 9, and 8. Uptake values were log10-transformed before analysis and analyzed using Type III two-way ANOVA with surgery and treatment as fixed factors, followed by Šidák-adjusted planned comparisons. Hepatectomy reduced glutamate uptake in saline-treated animals (adjusted *p* = 0.0040), whereas GUO increased uptake in hepatectomized animals (adjusted *p* = 0.0003). (**C**) GLAST immunocontent and (**D**) GLT-1 monomer immunocontent normalized to β-tubulin. Biological sample sizes were *n* = 4, 4, 3, and 3, respectively, for both Western blot analyses. Each value represents one animal; representative bands from one animal per group are shown. GLAST and GLT-1 monomer immunocontent were analyzed using Type III two-way ANOVA; no significant main effects or surgery × treatment interactions were observed. Data are presented in the original measurement scale as mean ± SEM.

Cortical Na^+^-dependent glutamate uptake was evaluated 24 h after surgery (Figure 4B). The biological sample sizes were 10, 5, 9, and 8 in the Sham + Saline, Sham + GUO, Hepatectomy + Saline, and Hepatectomy + GUO groups, respectively. Uptake values were log10-transformed before analysis. Two-way ANOVA showed no significant effect of surgery (F(1,28) = 0.24, *p* = 0.631, ηp^2^ = 0.008), a significant treatment effect (F(1,28) = 4.28, *p* = 0.0480, ηp^2^ = 0.133), and a significant surgery × treatment interaction (F(1,28) = 14.36, *p* = 0.0007, ηp^2^ = 0.339). In saline-treated animals, hepatectomy reduced glutamate uptake (Šidák-adjusted *p* = 0.0040). In hepatectomized animals, GUO increased glutamate uptake 2.50-fold relative to saline (95% CI 1.63–3.83; Šidák-adjusted *p* = 0.0003), whereas the treatment effect in sham animals was not significant (adjusted *p* = 0.452). GLAST and GLT-1 monomer immunocontent, normalized to β-tubulin, were evaluated in the Sham + Saline (*n* = 4), Sham + GUO (*n* = 4), Hepatectomy + Saline (*n* = 3), and Hepatectomy + GUO (*n* = 3) groups (Figure 4C,D). For GLAST, no significant effects of surgery (F(1,10) = 0.47, *p* = 0.509, ηp^2^ = 0.045), treatment (F(1,10) = 0.24, *p* = 0.632, ηp^2^ = 0.024), or surgery × treatment interaction (F(1,10) = 0.25, *p* = 0.630, ηp^2^ = 0.024) were detected. For the GLT-1 monomer, no significant effects of surgery (F(1,10) = 0.01, *p* = 0.924, ηp^2^ = 0.001), treatment (F(1,10) = 0.39, *p* = 0.547, ηp^2^ = 0.037), or surgery × treatment interaction (F(1,10) = 0.01, *p* = 0.937, ηp^2^ = 0.001) were observed.

## 4. Discussion

In this model of ALF-induced HE, GUO attenuated neurological deterioration and increased 72 h survival from 10.5% to 39.0%. These functional benefits were accompanied by attenuation of GFAP-associated astrocytic remodeling, lower CSF albumin, glutamate, and glutamine concentrations, and improved cortical Na^+^-dependent glutamate uptake, without significant changes in GLAST or GLT-1 monomer immunocontent. The convergence of survival, neurological, morphological, and neurochemical outcomes supports a protective role for GUO in ALF-induced HE and suggests that improved astrocyte-associated glutamate handling may contribute to its beneficial effects.

Hyperammonemia disrupts astrocytic metabolism, promotes intracellular glutamine accumulation and osmotic stress, and impairs regulation of the extracellular environment [17,18,24]. Consistent with this framework, subtotal hepatectomy increased GFAP-associated astrocytic complexity and reduced cortical Na^+^-dependent glutamate uptake. GUO attenuated the morphological alterations and improved uptake, supporting an effect on selected components of astrocyte-associated glutamatergic regulation. Together, these findings indicate that GUO improves a functional component of cortical glutamate handling while attenuating GFAP-associated astrocytic remodeling.

The dissociation between uptake function and transporter immunocontent is mechanistically relevant. Neither GLAST nor GLT-1 monomer immunocontent differed significantly among groups in the present Western blot analysis. Transporter function may be influenced by membrane localization, post-translational regulation, ionic gradients, and cellular energetic state without changes in total immunocontent. Therefore, the improved uptake observed after GUO may involve functional regulation of glutamate transporters, a possibility that should be examined in future studies [27,29]. The present study did not assess transporter localization or surface expression and did not quantify the higher-molecular-weight GLT-1-reactive bands. Consequently, changes in transporter trafficking, regulation, or oligomeric state remain hypotheses for future testing rather than demonstrated pathways of GUO action.

GFAP morphometry indicated that GUO attenuated reactive astrocytic remodeling. Primary and secondary process counts provide structural information but do not directly establish cell swelling, cell number, glutamate transport capacity, or metabolic flux. In particular, GS is central to the ATP-dependent amidation of glutamate and ammonia to form glutamine. GS expression and activity were not measured; therefore, uptake and subsequent intracellular metabolism must be interpreted separately. Future studies combining GFAP morphology with GS expression/activity, ammonia measurements, transporter surface localization, and isotope-tracing of the glutamate–glutamine cycle would provide a more complete mechanistic assessment.

Subtotal hepatectomy increased CSF glutamate, glutamine, and albumin, and GUO attenuated these elevations. Increased glutamate and glutamine are consistent with disruption of cerebral amino-acid handling during HE. Albumin is blood-derived, and CSF/serum albumin measures are commonly used to characterize blood-CSF barrier and CSF-flow-related function [52]. However, the present study did not include matched serum albumin, an albumin quotient, vascular tracer studies, or tight-junction histology. Accordingly, reduced CSF albumin after GUO is interpreted as attenuation of a barrier-related alteration, not direct proof that GUO restored blood–brain barrier permeability.

The protective effects of systemic GUO administration may involve complementary central and peripheral mechanisms. GUO or purine-related metabolites may reach the central nervous system and influence astrocytic signaling and glutamate transport, whereas peripheral effects on inflammatory, metabolic, vascular, or hepatic pathways may also reduce the neurotoxic burden associated with ALF. The present experimental design did not distinguish the relative contribution of these pathways. Future studies integrating plasma and CSF pharmacokinetics with route-specific administration will help clarify the principal sites and mechanisms underlying GUO action.

Current management of HE primarily targets ammonia production and disposal while also addressing the precipitating cause. Lactulose and rifaximin reduce gut-derived nitrogenous or neurotoxic burden, whereas L-ornithine-L-aspartate supports ammonia detoxification; branched-chain amino acids and zinc may be considered in selected nutritional or deficiency contexts [15,23,53]. Unlike these established approaches, the experimental rationale for GUO focuses on astrocyte-associated glutamate handling and cellular homeostasis. Other experimental strategies have targeted NMDA receptor signaling and glutamine synthetase activity [33,34,35]. These distinct mechanisms suggest a potentially complementary therapeutic rationale for GUO. Future comparative and combination studies should determine whether GUO can enhance the effects of established HE therapies.

Several considerations should be taken into account when interpreting these findings. No formal a priori sample-size or power calculation was performed. This represents a limitation of the study. Sample sizes were determined based on previous studies from our group using the same 92% subtotal-hepatectomy model, together with the expected mortality and the tissue requirements of each experimental endpoint. Because of the severity of the model, the final number of animals available differed among specific analyses. Only male rats were included, and future studies should determine whether the effects of GUO are influenced by sex. The present study was designed to evaluate neurological outcomes, survival, astrocytic morphology, CSF biomarkers, glutamate uptake, and transporter immunocontent, rather than to provide a comprehensive characterization of glutamate–glutamine flux, transporter membrane localization, GUO pharmacokinetics, or the relative contribution of central and systemic mechanisms. In addition, CSF albumin levels should be interpreted as an indicator of barrier-related alterations rather than as a direct measure of blood–brain barrier permeability. These considerations define the scope of the present findings and identify relevant directions for future mechanistic studies.

## 5. Conclusions

GUO attenuated neurological deterioration and improved survival in ALF-induced HE, in association with attenuated GFAP-associated astrocytic remodeling and CSF neurochemical alterations, as well as improved cortical Na^+^-dependent glutamate uptake. These effects occurred without significant changes in GLAST or GLT-1 monomer immunocontent, suggesting that the functional improvement in glutamate handling may not depend on changes in total transporter abundance. Overall, the present findings identify GUO as a promising experimental strategy for modulating astrocyte-associated glutamatergic dysfunction in acute hepatic encephalopathy.

## Data Availability

The original contributions presented in this study are included in this article. Further inquiries can be directed to the corresponding author.

## Supplementary Materials

The following supporting information can be downloaded at https://www.mdpi.com/article/10.3390/metabo16080587/s1: Table S1. Animal flow, endpoint-specific sample sizes, exclusions or unavailable observations, nested measurements, and experimental units.
