# Supplementary material for: Guanosine Attenuates Astrocyte-Associated Glutamatergic Dysregulation and Improves Survival in Acute Liver Failure-Induced Hepatic Encephalopathy
Source: Metabolites. 2026 Aug 18;16(8):587. doi: 10.3390/metabo16080587 (PMC13515678; doi:10.3390/metabo16080587)
Supplement: Supplementary file 1 [file metabolites-16-00587-s001.zip › metabolites-4454822-supplementary.pdf]

Supplementary Table S1

Guanosine attenuates astrocyte-associated glutamatergic dysregulation and improves survival in acute liver failure-induced hepatic encephalopathy

Animal flow, endpoint-specific sample sizes, exclusions or unavailable observations, nested measurements, and experimental units

Three animals died during surgery in the overall experimental series (sham, n = 1; hepatectomy, n = 2). The archived records did not permit these perioperative deaths to be assigned reliably to each endpoint-specific cohort. Therefore, unavailable historical values are identified as not separately recoverable and were not estimated.

Endpoint	Initial allocation or available cohort n SS/SG/HS/HG	Surgical or precollection losses	Postcollection exclusions or unavailable observations	Final biological n SS/SG/HS/HG	Mortality/survival information	Technical or nested measurements	Experimental unit
Neurological severity and survival	—/—/38/41	No additional surgical losses were entered in the survival file.	The grouped ordinal table has incomplete HS grade counts at 24 h (n = 36) and 48 h (n = 37); the survival analysis uses the complete time-to-event records.	—/—/38/41	By 72 h, 34/38 HS animals and 25/41 HG animals had died; survival was 10.5% and 39.0%, respectively.	Repeated neurological observations per animal; individual longitudinal identifiers were not retained in the archived grouped table.	Animal
GFAP primary processes	Not separately recoverable	Not separately recoverable	No exclusion was recorded for the analyzed values.	18/18/20/18	Not applicable	Standardized confocal observations from the same animal were averaged to one value; exact historical image/cell count was not retained.	Animal
GFAP secondary processes	Not separately recoverable	Not separately recoverable	Endpoint-specific image availability resulted in smaller SG and HS samples.	18/11/18/18	Not applicable	Standardized confocal observations from the same animal were averaged to one value; exact historical image/cell count was not retained.	Animal
CSF albumin	Not separately recoverable	Mortality and/or sample availability before the 24-h collection	No additional exclusion was recorded.	12/8/10/9	Not applicable	One animal-level chromatographic value; technical replicate count was not retained.	Animal
CSF glutamate	Not separately recoverable	Mortality and/or sample availability before the 24-h collection	No additional exclusion was recorded.	5/6/6/6	Not applicable	One animal-level chromatographic value; technical replicate count was not retained.	Animal
CSF glutamine	Not separately recoverable	Mortality and/or sample availability before the 24-h collection	No additional exclusion was recorded.	6/6/6/5	Not applicable	One animal-level chromatographic value; technical replicate count was not retained.	Animal
Na^+-dependent glutamate uptake	Not separately recoverable	Mortality and/or tissue availability before the 24-h collection	No additional exclusion was recorded.	10/5/9/8	Not applicable	Slice measurements were normalized to animal-level protein content; technical replicate count was not retained.	Animal
GLAST and GLT-1 monomer immunocontent	4/4/3/3 animals represented by 14 labeled lanes	None within the analyzed blot set	None; each labeled lane represented one animal.	4/4/3/3	Not applicable	One lane per animal; target signal normalized to the corresponding β-tubulin signal.	Animal

Abbreviations: SS, Sham + Saline; SG, Sham + GUO; HS, Hepatectomy + Saline; HG, Hepatectomy + GUO; CSF, cerebrospinal fluid; GFAP, glial fibrillary acidic protein; GLAST, glutamate-aspartate transporter; GLT-1, glutamate transporter 1.

Interpretive note: Endpoint-specific differences in n reflect mortality, sample or tissue availability, image availability, or the number of labeled Western-blot lanes recoverable from the archived records. No unavailable historical count was imputed.
